# Developing a stroke center in a Brazilian university hospital: implementation, challenges, and outcomes

**DOI:** 10.1055/s-0046-1817021

**Published:** 2026-03-19

**Authors:** Luciano Talma Ferreira, Vinicius Viana Abreu Montanaro, Márcia Silva Santos Neiva, Adriana Ferreira Barros Areal, Marcos Christiano Lange, Felipe von Glehn

**Affiliations:** 1 Universidade de Brasília, Hospital Universitário de Brasília, Brasília DF, Brazil.; 2 Rede SARAH de Hospitais de Reabilitação, Brasília DF, Brazil.; 3 Universidade Federal Fluminense, Programa de Pós-Graduação Stricto Sensu em Neurologia, Niterói RJ, Brazil.; 4 Universidade do Distrito Federal Professor Jorge Amaury Nunes Maia, Escola Superior de Ciências da Saúde, Curso de Medicina, Brasília DF, Brazil.; 5 Secretaria de Estado de Saúde do Distrito Federal, Brasília DF, Brazil.; 6 Universidade Federal do Paraná, Hospital de Clínicas, Curitiba PR, Brazil.; 7 Universidade de Brasília, Faculdade de Medicina, Brasília DF, Brazil.

**Keywords:** Stroke, Ischemic Stroke, Thrombolysis

## Abstract

**Background:**

Stroke remains the second leading cause of death worldwide and the leading cause in Brazil. The implementation of a stroke center (SC) has played a crucial role in improving outcomes, increasing both survival rates and functional recovery.

**Objective:**

To examine the experience of Hospital Universitário de Brasília of Universidade de Brasília's (HUB-UnB) SC, focusing on its performance indicators.

**Methods:**

The present observational, retrospective, and analytical study evaluated 19 patients diagnosed with acute ischemic stroke (AIS) who received intravenous thrombolysis with alteplase at the HUB-UnB SC between July 2021 and December 2024. Performance indicators were compared with national and international data.

**Results:**

The door-to-CT time was less than 25 minutes in 16 patients (84%), and the door-to-needle time was less than 60 minutes in 12 patients (63%). The mean National Institutes of Health Stroke Scale score significantly decreased from 12.4 at admission to 1.6 at discharge. Consequently, 76% of patients achieved a modified Rankin Scale score of 0 to 2. Complication rates were low, including 5.3% hemorrhagic transformation and 10.5% in-hospital mortality. No statistically significant differences were observed when comparing performance indicators with national and international benchmarks.

**Conclusion:**

The implementation of the HUB-UnB's SC proved feasible and effective in providing specialized care for AIS within a university hospital of the Unified Health System (Sistema Único de Saúde, SUS, in Portuguese). Despite operating only during weekday business hours, outcomes were comparable to those of high-income countries. Expansion to 24-hour operation, infrastructure improvements, recruitment of additional healthcare professionals, and greater integration with the SUS remain key challenges.

## INTRODUCTION

Stroke remains the second leading cause of death and the third leading cause of combined death and disability worldwide, with its global burden increasing significantly from 1990 to 2019, especially in lower-income and lower-middle-income countries. 1

According to the Brazilian Ministry of Health (BMH), stroke was the leading cause of death in Brazil in 2024. The age-adjusted incidence rates of stroke per 100 thousand inhabitants were 63 in Canoas, 106 in Joinville, 72 in Sertãozinho, and 96 in Sobral, which, having the lowest socioeconomic indexes, also showed the worst outcomes in lethality and functional status. 2 In 2012, the BMH published an ordinance establishing the criteria for hospital accreditation as a stroke center (SC) within the Unified Health System (Sistema Único de Saúde, SUS, in Portuguese), introducing the corresponding financial incentive and approving the Stroke Care Pathway. 3 Since then, the number of public hospitals with SCs in Brazil has steadily increased. 4 Stroke centers have been shown to improve patients' survival and independence. 5 However, in 2022, personal communication with neurologists from federal university hospitals (FUHs) affiliated with the Brazilian Hospital Services Company (Empresa Brasileira de Serviços Hospitalares, Ebserh, in Portuguese) indicated that 16 hospitals had neurologists, but only 3 had a certified SC. The state-owned company under the Brazilian Ministry of Education, Ebserh, was established in 2011 to manage FUHs. The network includes 51 hospitals associated with 36 federal universities, with 45 of them affiliated with Ebserh. 6

Since 2015, mechanical thrombectomy (MT) has been the standard of care for proximal large vessel occlusion (LVO) stroke, significantly improving patient outcomes. 7 In 2020, the RESILIENT trial, conducted in 12 Brazilian public hospitals, demonstrated that MT nearly tripled the chances of functional independence when performed within 8 hours of symptom onset, confirming its feasibility within SUS. 8 Based on these findings, ordinance published by the BMH in 2021 officially incorporated MT into SUS. 9 In 2023, a new ordinance established funding, designated the initial qualified SCs, and outlined guidelines for certifying additional SCs to perform MT. 10 However, Brazilian public hospitals face significant challenges, including deficits in infrastructure and human resources, limited access to essential imaging studies, a shortage of dedicated hospital beds, and an insufficient number of trained health professionals to provide quality care and train multidisciplinary teams. 11 These barriers further complicate the successful integration of MT into SUS.

Since 2012, intravenous thrombolysis (IVT) for acute ischemic stroke (AIS) within SUS of Distrito Federal (DF), where the nation's capital is located, has been exclusively available at Hospital de Base do Distrito Federal (HBDF), the sole public hospital in the region with a SC.

The current study presents the implementation and progressive development of a SC at Hospital Universitário de Brasília da Universidade de Brasília (HUB-UnB), a FUH managed by the Ebserh. Despite operating with limited resources and partial service hours, the HUB-UnB SC achieved outcomes comparable to national and international benchmarks. This initiative demonstrates the feasibility of establishing effective stroke care in a FUH and may serve as a scalable model for similar institutions throughout Brazil.

## METHODS

The current observational, retrospective, and analytical study included 19 patients diagnosed with AIS who received IVT at the HUB-UnB SC between July 2021 and December 2024. Data were obtained from electronic medical records and hospital databases.

Patients were eligible if acute stroke protocol (ASP) was activated, the diagnosis of AIS was confirmed, and IVT was administered. Exclusion criteria comprised cases in which the ASP was activated but the final diagnosis was not AIS, or when IVT was contraindicated.

Given the study's objective to document the initial experience of the HUB-UnB's SC, all eligible cases within the defined study period were included. Accordingly, the analysis was exploratory in nature and not based on a formal sample size calculation.

Performance indicators were compared with data from other Brazilian Public Hospitals' SCs—Hospital das Clínicas da Faculdade de Medicina de Botucatu da Universidade Estadual de São Paulo (HCFMB-UNESP), Complexo do Hospital de Clínicas da Universidade Federal do Paraná (CHC-UFPR), and the HBDF—as well as with international stroke registries, namely the Safe Implementation of Thrombolysis in Stroke-Monitoring Study (SITS-MOST) 12 and CASES, Carotid Artery Stenting during Endovascular treatment of acute ischemic Stroke (CASES) 13 studies. The HCFMB-UNESP and the CHC-UFPR were selected because they are university hospitals whose published data include the same key performance indicators required by the BMH. The HBDF was included because it is the only public hospital in DF with an accredited SC. All comparisons were performed using aggregated data extracted from published tables, without access to the original datasets; therefore, these analyses represent indirect comparisons that may be influenced by methodological heterogeneity across studies.

Neurological deficit severity was assessed using the National Institutes of Health Stroke Scale (NIHSS). Functional outcome at discharge was measured by the modified Rankin Scale (mRS). Stroke etiology was classified according to the Trial of Org 10172 in Acute Stroke Treatment (TOAST) criteria.

### Data collection

Data from the HUB-UnB's SC were obtained by accessing patient records. The variables evaluated were age; gender; origin; means of admission; comorbidities; onset-to-door (OTD) time; door-to-CT (DTCT) time; door-to-needle (DTN) time; onset-to-needle time or Delta T; DTCT time < 25 minutes; DTN time < 60 minutes; mRS at discharge; NIHSS score on admission, 1 hour and 24 hours post-thrombolysis, and at discharge; etiologic diagnosis—ischemic stroke (IS) with TOAST, transient ischemic attack (TIA), or stroke mimics; complications—hemorrhagic transformation, neurosurgical treatment, deep venous thrombosis (DVT), pressure ulcer (PU), pneumonia, urinary tract infection (UTI), stroke recurrence during hospital stay, hospital stay > 15 days, and in-hospital mortality; International Classification of Diseases—Tenth Revision (ICD-10) specific to the type of stroke at hospital discharge; DVT prophylaxis and antiplatelet therapy initiated by day 2; Antiplatelet therapy at discharge in non-cardioembolic IS; anticoagulant therapy at discharge in cardioembolic IS (atrial fibrillation); statin at discharge in atherothrombotic IS; rehabilitation plan and prophylaxis at discharge; and stroke patients admitted to the stroke unit.

Data from the HCFMB-UNESP and the CHC-UFPR's SCs were obtained indirectly from a table present in the study by Lange et al. 14

Data from the HBDF's SC and the SITS-MOST and CASES studies were also obtained indirectly from a table present in the study by Tosta et al. 15

### Statistical analysis

The variables were organized using a preconstructed electronic instrument developed in Microsoft Excel (Microsoft Corp.). Descriptive analysis of categorical variables was conducted by calculating absolute (n) and relative (-%) frequencies. Continuous quantitative variables were presented as mean ± standard deviation (SD) values. Statistical significance was set at a *p* -value < 0.05.

The normality of continuous variables was assessed using the Shapiro-Wilk test and by visual inspection of histograms and normal probability (quantile-quantile, Q-Q) plots. Since most variables did not follow a normal distribution, non-parametric tests were applied, which are more appropriate for small samples and non-normally distributed data.

Comparisons between independent samples were performed using the Mann-Whitney U test, a non-parametric alternative to Student's t-test for numerical variables.

To assess differences in frequency distributions, the goodness-of-fit test, a non-parametric method used to determine whether an observed distribution aligns with an expected or reference distribution, was applied. Specifically, the Chi-squared test was employed to evaluate the goodness-of-fit between observed and expected distributions for categorical variables. The statistical analysis of differences in certain categorical variables was not feasible due to the occurrence of frequencies at 0% or 100%. Similarly, the analysis of differences in some continuous quantitative variables could not be conducted due to the lack of SDs.

### Ethical statement

The Ethics Committee of the Faculty of Medicine of Universidade de Brasília approved the study (CAAE: 68068523.4.0000.5558), and all participants signed the informed consent form (ICF). The ICF was required because the original research protocol included a prospective analysis designed to collect additional follow-up data on return to work, quality of life, and sexual function. Only aggregated, publicly available data from other hospitals were used for external comparisons; therefore, separate ethical approval was not required for those datasets.

## RESULTS

Between July 2021 and December 2024, the ASP was activated for 71 patients, of whom 25 (35%) were transported by the Urgent Mobile Care Service (Serviço de Atendimento Móvel de Urgência, SAMU, in Portuguese). Among these patients, 15 (60%) were diagnosed with AIS and subsequently received IVT. In contrast, of the 46 patients not transported by SAMU, only 4 (8.7%) received IVT, including 2 cases of in-hospital stroke (IHS). Findings refer to a cohort of 19 patients diagnosed with AIS who received IVT.

### Demographic characteristics, comorbidities and risk factors

Most patients (79%) were female, with a mean age of 62.9 ± 15 years, and 85% were from outside the central health region of DF. The 5 most common comorbidities and risk factors were hypertension (73%), overweight/obesity (68%), dyslipidemia (63%), active smoking (42%), and prior stroke (42%) ( Table 1 ).

**Table 1:** Demographic characteristics, comorbidities and risk factors, clinical data, performance indicators, complications, and etiological diagnosis of a cohort of 19 patients diagnosed with acute ischemic stroke who received intravenous thrombolysis

			N (%)
Gender	Female		15–19 (79%)
	Male		4–19 (21%)
Health region of origin	Others		16–19 (84%)
	Central		3–19 (16%)
Comorbidities and risk factors	Systemic arterial hypertension		14–19 (73%)
	Overweight or obesity		13–19 (68%)
	Dyslipidemia		12–19 (63%)
	Active smoking		8–19 (42%)
	Previous stroke		8–19 (42%)
	Prediabetes		6–19 (32%)
	Type 2 diabetes mellitus		5–19 (26%)
	Alcoholism		3–19 (16%)
	Other cardiac arrhythmias		2–19 (11%)
	Atrial fibrillation		1–19 (5%)
			Mean ± SD
Other variables	Age		62.6 ± 15
	NIHSS on admission		12.4 ± 7.5
	NIHSS after 1 hour		6.4 ± 5.9
	NIHSS after 24 hours		3.6 ± 6.2
	NIHSS at discharge		1.6 ± 3.6
	Door-to-CT time		17.8 ± 16.1
	Door-to-needle time		55.8 ± 34.5
	Onset-to-needle time (delta T)		144.4 ± 57.1
	Door-to-CT time < 25 minutes		16–19 (84%)
	Door-to-needle time < 60 minutes		12–19 (63%)
			N (%)
Functional outcome	mRS	0, 1 or 2	13–17* (76%)
		3	1–17* (6%)
		4	0 (0%)
		5	1–17* (6%)
		6	2–19 (10.5%)
Complications	Hemorrhagic transformation		1–19 (5.26%)
	Need for neurosurgical intervention		0 (0%)
	Pulmonary thromboembolism		1–19 (5.26%)
	Venous thromboembolism		0 (0%)
	Infection		1–19 (5.26%)
	Stroke recurrence during hospitalization		0 (%)
	Length of stay > 15 days		1–16** (6.25%)
	Hospital mortality		2–19 (10.53%)
Etiological diagnosis	Stroke etiological subtype according to TOAST	1	5–19 (26%)
		2	2–19 (10%)
		3	0 (0%)
		4	1–19 (5%)
		5	6–19 (31%)
	Stroke mimics		4–19 (21%)
	Transient ischemic attack		1–19 (5%)

Abbreviations: mRS, modified Rankin Scale; NIHSS, National Institutes of Health Stroke Scale; TOAST, A system for categorizing ischemic stroke *subtypes, primarily based on etiology, developed for the Trial of Org 10172 in Acute Stroke Treatment* .

Notes: *Exclusion of 2 patients transferred to another hospital within 24 hours; **Exclusion of 2 patients transferred to another hospital within 24 hours and 1 patient who left the hospital within 48 hours.

### Performance indicators

The DTCT time was shorter than 25 minutes in 16 patients (84%), with a mean of 17.8 ± 16.1 minutes. The DTN time was less than 60 minutes in 12 patients (63%), with a mean of 55.8 ± 34.5 minutes. The delta T had a mean of 144.4 ± 57.1 minutes ( Table 1 ).

The delta T was not significantly shorter in IHS patients compared to those transported by SAMU ( *p* -value: 0.50). However, DTCT and DTN times were significantly shorter among patients transported by SAMU compared to those with IHS ( *p* -value of both: 0.04) ( Table 2 ).

**Table 2:** Comparison of treatment times between patients transported by SAMU and those with in-hospital stroke

Comparison		Mean	SD	N	*p* -value
1. DTCT	IHS	51.5	± 12	2	0.04*
	SAMU	14.6	± 11.7	15	
2. DTN	IHS	115	± 57.9	2	0.04*
	SAMU	46.7	± 18.3	15	
3. Delta T	IHS	115	± 57.9	2	0.50
	SAMU	148.5	± 46.5	15	

Abbreviations: Delta T, onset-to-need time; DTCT, door-to-CT time; DTN, door-to-needle time; IHS, in-hospital stroke patients; N, number of patients; SAMU, patients transported by SAMU; SD, standard deviation.

Notes: Statistical analysis was performed using the Mann-Whitney U test. *Statistically significant.

### Clinical outcomes and complications

The NIHSS score demonstrated a progressive and significant decline from admission to hospital discharge. The mean score decreased from 12.4 ± 7.5 upon admission to 6.4 ± 5.9 1 hour after IVT ( *p* -value: 0.01), 3.6 ± 6.2 24 hours after IVT ( *p* -value: < 0.001), and 1.6 ± 3.6 upon discharge ( *p* -value: < 0.001). Consequently, 76% of patients achieved a favorable outcome, defined as a mRS score of 0 to 2. Patients experienced low complication rates, including 5.26% hemorrhagic transformation and 10.53% in-hospital mortality ( Tables 1 3 ).

**Table 3:** Comparison of NIHSS at admission with NIHSS at 1 hour after IVT, NIHSS at 24 hours after IVT, and NIHSS at discharge

Comparison		Mean	SD	N	*p* -value
1	NIHSS at admission	12.4	± 7.5	19	0.010*
	NIHSS at 1 hour after IVT	6.4	± 5.9	19	
2	NIHSS at admission	12.4	± 7.5	19	< 0.001*
	NIHSS at 24 hours after IVT	3.6	± 6.2	19	
3	NIHSS at admission	12.4	± 7.5	19	< 0.001*
	NIHSS at discharge	1.6	± 3.6	19	

Abbreviations: IVT, ibntravenous thrombolysis; N, number of patients; NIHSS, National Institutes of Health Stroke Scale; SD, standard deviation.

Notes: Statistical analysis was performed using the Mann-Whitney U test; *Statistically significant.

### Etiological diagnosis

Among 14 patients with confirmed diagnosis of AIS, 6 (43%) had an undetermined etiology, 5 (36%) had a large-artery atherosclerosis etiology, 2 (14%) had a cardioembolic etiology, and 1 (7%) had another determined etiology. Of the 6 cryptogenic cases, 4 did not complete an etiological workup due to insufficient time: 2 were transferred within 24 hours, 1 was discharged within 48 hours, and 1 died on the 4th day of hospitalization. Four patients (21%) were diagnosed with stroke mimics, including epileptic seizures (2), migraine with aura (1), and functional neurological disorder (1). One patient (5%) was diagnosed with a TIA. The diagnoses of stroke mimics and TIA were made during follow-up at the neurovascular outpatient clinic after reviewing the history and complementary exams ( Table 1 ).

The majority (89%) of patients underwent head and neck computed tomography angiography, identifying LVO in 5 patients. However, none received MT due to its unavailability at HUB-UnB and within the SUS of the DF.

### Comparisons with other stroke centers

When compared with two other Brazilian UHs (HCFMB-UNESP and CHC-UFPR), HUB-UnB demonstrated a shorter DTCT time and lower or comparable rates of complications (DVT, pneumonia, UTI, and PU). It also showed a lower mortality rate than CHC-UFPR, as well as higher or equivalent proportions of stroke patients admitted to the stroke unit, antiplatelet therapy on day 2, antiplatelet therapy at discharge in non-cardioembolic ischemic stroke, anticoagulant therapy at discharge in cardioembolic ischemic stroke, statin use at discharge in atherothrombotic ischemic stroke, and documented rehabilitation planning and prophylaxis at discharge. Conversely, HUB-UnB presented a longer DTN time, a higher mortality rate than HCFMB-UNESP, and lower rates of DVT prophylaxis on day 2 and specification of stroke etiology according to ICD-10 at discharge. No statistically significant differences were observed among the variables, and statistical analysis of some indicators was not feasible due to absolute (0% or 100%) frequencies. ( Table 4 )

**Table 4:** Comparison of the KPIs of the HUB-UnB with those of two other Brazilian university hospitals

Variable	HUB-UnB	HCFMB-UNESP	CHC-UFPR
Number of patients	19	172	117
DTCT < 25 minutes	84%	27% ( *p* -value 0.12)	83% ( *p* -value 0.98)
DTN < 60 minutes	63%	75% ( *p* -value 0.80)	66% ( *p* -value 0.95)
DVT prophylaxis on day 2	75%	92% ( *p* -value 0.69)	88% ( *p* -value 0.76)
Antiplatelet therapy on day 2	100%	97%	94%
Antiplatelet therapy at discharge in non-cardioembolic IS	100%	75%	100%
Anticoagulation therapy at discharge in cardioembolic (atrial fibrillation) IS	100%	94%	87%
Statin at discharge in atherothrombotic IS	100%	100%	88%
Plan of rehabilitation and prophylaxis at discharge	100%	96%	87%
Percentage of stroke patients admitted to the stroke unit	95%	94% ( *p* -value 0.96)	84% ( *p* -value 0.61)
DVT	0	0.6%	0
Pneumonia	0	13%	6%
UTI	1%	11% ( *p* -value 0.31)	6% ( *p* -value 0.62)
PU	0	0.6%	1%
Mortality rate	10.5%	8% ( *p* -value 0.94)	14% ( *p* -value 0.91)
Specific ICD-10 to stroke etiology at discharge	0	24%	64%

Abbreviations: CHC-UFPR, Complexo do Hospital de Clínicas da Universidade Federal do Paraná; DTCT, door-to-CT time; DTN, door-to-needle time; DVT, deep venous thrombosis; HCFMB-UNESP, Hospital das Clínicas da Faculdade de Medicina de Botucatu da Universidade Estadual de São Paulo; HUB-UnB, Hospital Universitário de Brasília da Universidade de Brasília; ICD, international code diagnosis; IS, ischemic stroke; KPI, key performance indicator; PU, pressure ulcer; UTI, urinary tract infection.

Notes: Data from the HCFMB-UNESP and the CHC-UFPR strokes centers were obtained indirectly from a table present in the study by Whalgren et al. 12 Statistical analysis was performed using the Chi-squared goodness-of-fit test. The statistical analysis of differences in certain categorical variables was not feasible due to the occurrence of frequencies at 0% or 100%.

When compared with HBDF and the SITS-MOST and CASES studies, HUB-UnB demonstrated a lower proportion of male patients and a lower rate of hemorrhagic transformation, as well as shorter or comparable DTN time and delta T. It also showed a higher percentage of patients with significant neurological improvement (NIHSS decrease ≥ 4 points within 24 hours), a greater frequency of favorable functional outcomes at discharge, and lower in-hospital mortality rates than HBDF and the SITS-MOST study. Moreover, the OTD time was shorter than that observed at HBDF. Conversely, HUB-UnB exhibited a longer OTD time than the SITS-MOST study and a higher mortality rate than the CASES study. A statistically significant difference ( *p* < 0.01) was found in the proportion of male patients, while no significant differences were observed in the other variables. Statistical analysis of some indicators was not feasible due to the absence of standard deviations ( Table 5 ).

**Table 5:** Comparison of the HUB-UnB variables with those from HBDF and findings from the SIST-MOST and CASES studies

Variable	HUB-UnB	HBDF	SITS-MOST	CASES
Number of patients	19	32	6,483	1,135
Study period (months)	36	12	NR	30
Mean Age (years)	62	59	68	73
Variation	33–81	31–86	59–75	63–80
Gender (% male)	21	59 ( *p* -value 0.00)	60 ( *p* -value 0.00)	55 ( *p* -value 0.00)
NIHSS variation	2–29	3–24	8–17	9–19
Onset-to-door time (min)	106 (55–175)	118 (11–228)	72 (NR)	NR
Door-to-needle time (min)	51 (11–156)	89 (32–244)	68 (NR)	85 (60–109)
Onset-to-needle time (min)	140 (74–249)	195 (60–270)	140 (115–165)	155 (130–175)
Significant improvement in NIHSS ^+^	70.5%	50% ( *p* -value 0.65)	55% ( *p* -value 0.73)	NR
Favorable result at discharge (mRS 0-2)	76%	44% ( *p* -value 0.45)	39% ( *p* -value 0.39)	NR
Hemorrhagic transformation	5%	6% ( *p* -value 0.96)	17% ( *p* -value 0.58)	28.9% ( *p* -value 0.27)
Mortality	10.5%	13% ( *p* -value 0.94)	11.30% ( *p* -value 0.98)	3.6% ( *p* -value 0.82)

Abbreviations: CASES, Carotid Artery Stenting during Endovascular treatment of acute ischemic Stroke; HBDF, Hospital de Base do Distrito Federal; HUB-UnB, Hospital Universitário de Brasília da Universidade de Brasília; Min, minute; mRS, modified Rankin Scale; NIHSS, National Institutes of Health Stroke Scale; NR, not reported; SITS-MOST, Safe Implementation of Thrombolysis in Stroke-Monitoring Study.

Notes: Data from the HBDF's stroke center and the SITS-MOST and CASES studies were obtained indirectly from a table present in a reference. 15 Statistical analysis was performed using the Chi-squared goodness-of-fit test. The statistical analysis of differences in certain categorical variables was not feasible due to the occurrence of frequencies at 0% or 100%. The analysis of differences in continuous quantitative variables could not be conducted due to the lack of standard deviations. ^+^ Decrease of ≥ 4 points in 24 hours.

## DISCUSSION

The current study demonstrated that the implementation of the HUB-UnB SC was feasible and effective in providing specialized care for AIS within a FUH of SUS. The DTCT and DTN times met established quality indicators, and patients treated with IVT showed significant neurological improvement and favorable functional outcomes. Complication rates were low, and no significant differences were observed when comparing performance indicators with national and international benchmarks, underscoring the quality and safety of acute stroke management at HUB-UnB, despite operational limitations.

The Global Burden of Disease 2019 data show that stroke incidence and prevalence are higher in women than in men, 1 particularly among those under 45 years of age, in whom pregnancy-related conditions further increase the long-term risk of stroke. 16 In our cohort, women accounted for 79% of cases, reinforcing this epidemiologic pattern.

Stroke also disproportionately affects socioeconomically deprived populations, who face higher exposure to conventional vascular risk factors and reduced access to high-quality acute and rehabilitation care. 17 In the present study, most patients (84%) came from health regions outside the central area of DF, which is characterized by higher household income, suggesting that most originated from areas with lower socioeconomic status.

Consistent with the INTERSTROKE study, 18 our results showed a high prevalence of modifiable risk factors—hypertension, overweight or obesity, and smoking—which were present in 73%, 68%, and 42% of patients, respectively. Additionally, 42% had a prior stroke, highlighting the need for more effective secondary prevention strategies, as the risk of recurrence can reach 30% within 5 years. 19

Cases of IHS exhibited shorter delta T due to the absence of prehospital delays, but longer in-hospital treatment intervals, with mean DTCT and DTN times of 51.5 and 115 minutes, respectively, compared with 14.6 and 46.7 minutes for patients transported by SAMU. Complication rates were low in both groups, and the prolonged DTCT and DTN times did not translate into worse outcomes for IHS patients. These delays likely stem from slower neurologist notification and logistical barriers to treating already hospitalized patients when SC beds are unavailable. 20 Updating ASP to allow any staff member to activate it upon detecting new neurological deficits, 21 and including unit heads in the communication network, could improve coordination and response times.

Stroke mimics, which account for up to 25% of suspected stroke cases, 22 were identified in 21% of IVT-treated patients in our cohort, with no complications. This aligns with multicenter data showing that thrombolysis in mimics is generally safe, with hemorrhage rates far lower than in true ISs. 23 These findings reinforce the need to prioritize minimizing treatment delays over exhaustive diagnostic confirmation that may compromise timely reperfusion.

The HUB-UnB SC currently operates only on weekdays from 7 a.m. to 5 p.m. due to the limited number of neurologists. Implementing a 24/7 on-site shift system for neurologists in the ED and establishing a continuous education program can help reduce DTN time, increase the percentage of DVT prophylaxis on the second day, and ensure proper specification of stroke etiology according to ICD-10 at discharge. Improving general supportive care and managing acute complications are key to reducing mortality, while decreasing OTD times requires strengthening public awareness of stroke symptoms and optimizing SAMU response. 24

At the system level, UHS accreditation for SCs requires 24/7 emergency coverage, interpreted as an “open-door” ED. However, most FUHs operate “closed-door” EDs that admit only pre-referred patients, including HUB-UnB, which has limited its accreditation. In 2022, communication with Ebserh-affiliated neurologists revealed that 9 of 16 FUHs with neurologists had EDs, all closed-door.

Telestroke offers a feasible strategy to expand access to acute stroke care. Evidence shows that telestroke increases thrombolysis rates without compromising safety or efficacy. 25 26 Although no structured program exists within SUS, some public hospitals rely on private providers. Ebserh could instead develop a national telestroke network to support its FUHs with remote neurological expertise.

Despite its contract with the FD Health Department since 2017, the HUB-UnB has not been fully integrated into the Stroke Care Pathway of DF, resulting in a small number of patients transported by SAMU (25) and treated with IVT (19) between 2021 and 2024—far fewer than the 669 IVT cases recorded at HBDF between 2012 and 2020, including 178 in 2019 alone. This limited integration remains a major barrier to scaling stroke care within the FUHs.

The current study has limitations, including its observational design, small sample size, and restriction to AIS patients who underwent IVT, which prevented assessment of contraindications among the remaining 73% of cases. Comparative analyses between treated and untreated patients could yield valuable insights to refine treatment protocols. Additionally, comparisons with other centers relied on aggregated published data rather than individual-level datasets, introducing potential bias. Nonetheless, presenting the unadjusted performance indicators of HUB-UnB alongside national and international data provides valuable context and supports future multicenter studies using harmonized data.

Overall, the present study demonstrates that establishing a specialized SC within the FUHs of SUS is both feasible and effective, even under resource limitations. Persistent challenges include expanding to 24/7 operation, improving infrastructure, recruitment of additional healthcare professionals, and achieving formal accreditation. Full integration into the SUS of DF, combined with continuous multidisciplinary training, remain essential priorities. The HUB-UnB experience underscores the potential of FUHs to expand equitable access to IVT and enhance the quality of stroke care across Brazil.
